# The Influence of Early Nutrition on Neurodevelopmental Outcomes in Preterm Infants

**DOI:** 10.3390/nu15214644

**Published:** 2023-11-01

**Authors:** Rita C. Silveira, Andrea L. Corso, Renato S. Procianoy

**Affiliations:** Department of Pediatrics, Newborn Section, Hospital de Clínicas de Porto Alegre, Universidade Federal do Rio Grande do Sul, Porto Alegre 3452925, RS, Brazil

**Keywords:** preterm, early nutrition, macronutrients, micronutrients, human milk, human milk oligosaccharides, probiotics

## Abstract

Premature infants, given their limited reserves, heightened energy requirements, and susceptibility to nutritional deficits, require specialized care. Aim: To examine the complex interplay between nutrition and neurodevelopment in premature infants, underscoring the critical need for tailored nutritional approaches to support optimal brain growth and function. Data sources: PubMed and MeSH and keywords: preterm, early nutrition, macronutrients, micronutrients, human milk, human milk oligosaccharides, probiotics AND neurodevelopment or neurodevelopment outcomes. Recent articles were selected according to the authors’ judgment of their relevance. Specific nutrients, including macro (amino acids, glucose, and lipids) and micronutrients, play an important role in promoting neurodevelopment. Early and aggressive nutrition has shown promise, as has recognizing glucose as the primary energy source for the developing brain. Long-chain polyunsaturated fatty acids, such as DHA, contribute to brain maturation, while the benefits of human milk, human milk oligosaccharides, and probiotics on neurodevelopment via the gut-brain axis are explored. This intricate interplay between the gut microbiota and the central nervous system highlights human milk oligosaccharides’ role in early brain maturation. Conclusions: Individualized nutritional approaches and comprehensive nutrient strategies are paramount to enhancing neurodevelopment in premature infants, underscoring human milk’s potential as the gold standard of nutrition for preterm infants.

## 1. Introduction

Preterm infants constitute a diverse population with unique nutritional requirements that necessitate individualized approaches tailored to their clinical status and degree of prematurity. The overarching goal is to enhance neurodevelopmental outcomes, all while acknowledging the nutritional challenges posed by preterm birth. The brain, being the most metabolically active organ in premature infants, demands a substantial supply of nutrients for optimal growth and functional development. While all nutrients play vital roles, some exert a more pronounced impact, particularly during the critical period spanning from 24 to 52 weeks post-conceptional age. This timeframe represents a pivotal window for neurodevelopment, encompassing the development of essential structures such as white and gray matter, cell replication, neurogenesis, neuronal and cerebral white matter differentiation, cell migration, myelination, and synaptogenesis, among other intricate processes [1,2,3].

The abrupt cessation of placental nutrient delivery places preterm infants at a heightened risk for restricted postnatal growth. These infants are born with limited reserves of essential nutrients, exhibit compromised thermoregulation, and have elevated energy requirements. The primary challenge lies in identifying the key nutrients crucial for optimal brain development, considering factors like the neonate’s gestational age, underlying morbidities, enteral versus parenteral feeding capacities, and growth requirements. The early initiation of aggressive enteral feeding alongside appropriate parenteral support holds the potential to enhance the growth and overall development of very low-birth-weight and extremely premature infants. Conversely, excessive early nutritional support in preterm infants may lead to alterations in body composition and subsequently increase the risk of obesity and chronic non-communicable diseases later in life [1,4,5].

Recently, the concept of the microbiota-gut-brain axis has gained prominence, shedding light on the intricate interplay between the intestinal microbiota and the central nervous system. Accumulating evidence underscores the significance of the gut microbiome in this bidirectional communication system, constituting a complex network that modulates immune, gastrointestinal, and central nervous system functions [6,7].

The term “gut microbiome” encompasses the complex ecosystem of bacteria inhabiting the intestine, including their genes, proteins, and metabolites. Investigations into the interaction between the intestinal microbiota and the central nervous system have unveiled critical developmental windows, especially in the vulnerable preterm population. Manipulating the intestinal microbiota through prebiotic and probiotic supplementation presents a promising avenue for improving neurobehavioral outcomes in preterm infants. For instance, the absence of Bifidobacterium during the first month of life in preterm infants has been associated with delayed neurodevelopment in early childhood. The administration of Bifidobacterium strains may foster optimal neurocognitive development in these vulnerable children [6,7,8].

Hence, any nutritional strategy designed to mitigate initial weight loss and foster brain growth holds the potential to significantly enhance neurodevelopmental outcomes in preterm infants, particularly those born extremely prematurely, who traditionally face a higher risk of adverse outcomes. This review aims to provide a comprehensive overview of the current understanding of nutritional interventions, from neonatal to post-hospital discharge and follow-up, that can positively influence neurodevelopment. Specific nutrients, such as amino acids and lipids, nutritional supplements, and dietary practices, including breastfeeding and its undeniable benefits, are addressed. In this review, we address neuro-nutrition as a key concept of brain health, where each nutrient has its specific function in the developing brain of preterm infants (Table 1).

## 2. Data Sources

Data sources: PubMed and MeSH and keywords: preterm, early nutrition, macronutrients, micronutrients, human milk, human milk oligosaccharides, probiotics AND neurodevelopment or neurodevelopment outcomes. Recent articles were selected according to the authors’ judgment of their relevance.

## 3. Amino Acids

The literature extensively details the cognitive benefits of early, aggressive amino acid supplementation. Very low-birth-weight preterm infants who receive higher protein and energy intake during their first week of life exhibit improved IQ scores and reduced developmental delays at 18 months of age. This positive outcome is often accompanied by adequate head-circumference catch-up growth or accelerated head circumference growth. Additionally, very low-birth-weight premature infants with substantial head circumference growth tend to demonstrate better cognitive outcomes [11,12,13].

Despite the structural and functional immaturity of the intestines, which may hinder enteral nutrition progress, early initiation of enteral feeding is strongly recommended. Cormack and colleagues established a positive correlation between enteral protein intake in the first two weeks of life and cognitive and motor subscale scores on the Bayley III scale at 18 months of corrected age [14].

Evidence indicates that nutritional deficits at 28 days after birth are negatively associated with neurodevelopment at 3 months of age. Furthermore, studies have shown that higher energy and protein intake during the first week after birth are linked to improved neurodevelopment at 18 months of corrected age in premature infants [15,16].

A Cochrane review supports the early provision of amino acids in parenteral nutrition, leading to increased head circumference growth and reduced postnatal growth failure at discharge. The number needed to treat for an additional beneficial outcome is 7 (NNT = 7) [17]. Subgroup analyses have indicated a significant reduction in postnatal growth failure at discharge for preterm infants receiving high amino acid intake in parenteral nutrition (>2 to ≤3 g/kg/d). However, the impact on neurodevelopmental outcomes remains inconclusive [17].

In practice, the recommended target dose of amino acids in parenteral solutions remains under discussion. Suggestions for premature infants range from 3 g/kg/day to 3.5 g/kg/day of amino acids. There is no evidence to support better neurodevelopment with a maximum dose of 4 g/kg/day [18].

Determining the optimal amino acid amount in enteral nutrition for formula-fed low-birth weight preterm infants remains a subject of debate. Protein intake should suffice to achieve normal growth without causing undesirable effects such as acidosis, uremia, or elevated circulating amino acid levels. Simultaneously, it should aim to enhance head circumference growth and subsequently improve developmental outcomes. A Cochrane meta-analysis comparing low amino acid intake (<3 g/kg/d) versus high amino acid intake (3.0 to 4.0 g/kg/d) in formulas found that high protein intake (≥3.0 g/kg/d and <4.0 g/kg/d) accelerated the weight gain of preterm infants, although methodological limitations hindered a conclusive determination of its favorability [19].

Although breast milk is considered the gold standard for nutrition, its protein concentration may not meet the needs of preterm infants due to limitations in enteral volume supply. Therefore, breast milk is often supplemented with proteins and other nutrients [3]. However, determining the optimal concentration of commercially available proteins remains inconclusive despite numerous studies and reviews aiming to enhance nutritional performance for neurodevelopment [2,13,14,20].

Kumar et al., previously reinforced the role of individualized human milk fortification through two methods: target and adjustable fortification. Target fortification involves analyzing the protein content of human milk and adding additives based on the baby’s specific nutritional requirements, pre-defined by the care team. In adjustable fortification, protein intake is periodically adjusted based on the child’s metabolic response assessed by laboratory tests, making it more suitable for stable premature babies and practical, as it does not require a human milk analysis. Fortification significantly impacts head circumference growth and neurodevelopment [3].

A systematic review encompassing nine studies with a total of 861 premature infants found that a high protein concentration (≥1.4 g/100 mL) in human milk additives derived from bovine milk increased in-hospital weight gain compared to a moderate protein concentration (≥1 g < 1.4 g/100 mL). However, there was insufficient evidence to assess the impact of protein concentration on adverse effects or long-term outcomes like neurodevelopment [20].

Currently, recommendations suggest providing premature babies with a gestational age below 32 weeks at birth with 3.5 to 4.5 g/kg of proteins per day. As enteral supply improves, amino acids in parenteral nutrition should be reduced to ensure that the total protein intake does not exceed 4.5 g/kg per day (combined enteral and parenteral). Limited evidence suggests that a protein-to-calorie ratio of 30 to 40 kcal per gram can maximize protein synthesis and positively impact the neurodevelopment of premature infants [21].

It is worth noting that the type of amino acid offered may be more influential than the quantity in terms of neurocognition. Low taurine levels in the neonatal period of premature infants negatively affect neurological development, highlighting the advantage of breast milk, which contains high levels of taurine, the most abundant free amino acid in human milk, along with glutamate [22]. Taurine’s beneficial role primarily involves auditory and visual development and is frequently supplemented in infant formulas [21].

In the developing brain, GABA and glutamate play essential roles in neurotransmission, neuronal migration, dendrite and synapse formation, and neural circuit organization. The abundance of glutamate, relative to other acids, in human milk reinforces its neurocognitive benefits [23].

## 4. Glucose

Glucose serves as the primary energy source for the brain and nervous system, with the human brain consuming about 20% of the total body’s glucose in normal circumstances. In cases of limited glucose availability, the brain can utilize lactate and ketone bodies as alternative energy sources. Lactate sustains brain activity during glucose deprivation and supports several neuronal functions [24].

Owing to their premature birth, preterm infants have inadequate energy reserves, as glycogen accumulation in their bodies is insufficient. Recommended daily energy intake for these infants ranges from 10 to 130 kcal/kg/day to match intrauterine growth rates. Carbohydrates, primarily in the form of glucose, are the main energy source (4 kcal/gram) for newborns. Premature infants exhibit much higher rates of glucose synthesis (6–8 mg/kg/min) compared to full-term neonates (3–5 mg/kg/min) [1,2,11].

Studies have indicated that higher energy and protein intake during the first month after birth in preterm infants are associated with head circumference growth and improved cognitive outcomes in adolescence. Furthermore, enteral calorie supply appears to be more effective than parenteral delivery. For instance, a recent randomized clinical trial comparing early Parenteral Nutrition (PN) with enhanced early PN did not show differences in neurodevelopment but revealed that the enteral intake of calories and proteins in the first week was associated with improved processing speed in evoked potential tests [2,18,25].

The enhanced nutritional protocol entails initiating total parenteral nutrition with a fluid volume of 80 mL/kg/day to provide 4 g/kg/day of amino acids and a minimum glucose infusion rate of 5.5 mg/kg/min. Glucose infusion is increased by 1.5 mg/kg/min daily during the first week, up to a maximum of 12–14 mg/kg/min. Lipids are administered at 2.5–3.5 g/kg/day, and amino acids are maintained at 4 g/kg/day until enteral feeding reaches an average volume of 100 mL/kg/day [2].

Hyperglycemia for more than 5 days in premature infants under 32 weeks of gestational age has been associated with a lower lean mass at 4 months and worse neurodevelopment at 12 months of corrected age. This relationship may be linked to reduced glucose infusion rates in the first week to manage hyperglycemia [26].

## 5. Lipids

Premature birth is associated with a deficiency of long-chain polyunsaturated fatty acids (LCPUFAs), including docosahexaenoic acid (DHA) and arachidonic acid (ARA). This deficiency persists due to the ineffective conversion of precursor fatty acids, lower fat stores, and limited nutritional supply of DHA and ARA. LCPUFAs are essential for neurodevelopment, normal vision, and protection against complications like Bronchopulmonary Dysplasia, Retinopathy of Prematurity, and Necrotizing Enterocolitis [18,24].

LCPUFAs also play a crucial role in moderating the effects of hypoxia, inflammation, infection, thrombosis, and oxidative damage in key organs like the lungs, brain, and retina. DHA influences the structure of neuronal membranes, synaptogenesis, and myelination. Supplementation with DHA in formula for premature babies has been associated with improved electroretinogram activity, resulting in better visual acuity and overall development in the short term [24,27].

Higher energy and lipid supply in the first two weeks of life have been linked to a reduced risk of severely abnormal MRI findings at full-term age, especially in gray matter, cortex, and cerebellum. Enteral lipids appear to be more effective than intravenous lipid emulsions in this regard. The variable LCPUFA content in different lipid emulsions may account for the differences in outcomes. Additionally, a greater growth of subcortical structures, the cerebellum, and the entire brain, as well as accelerated microstructural maturation of white matter, have been observed in preterm infants under 30 weeks who received more extensive lipid supplementation in enteral nutrition. Enhanced energy and lipid intake may mitigate the adverse impact of respiratory morbidity on brain development, resulting in improved neurodevelopment at 18 months corrected age [5,16,28].

Enteral lipid supplementation has generated conflicting results. While early LCPUFA supplementation in preterm infants has shown positive impacts on early childhood psychomotor neurodevelopment and visual acuity, its effects on long-term global intelligence quotient (IQ) have been less significant [24,29]. Meta-analyses have indicated improved neurodevelopment in premature infants receiving LCPUFA supplementation, as assessed by the Bayley scales between one and three years of age. However, supplementation during lactation accelerates neurodevelopment, with no subsequent changes in developmental outcomes, leading to some frustration among researchers [30,31,32].

Current recommendations for follow-on formulas suggest that they should contain LCPUFAs at levels similar to those of human milk [33]. It is essential to recognize that nutritional management often fails to provide sufficient preformed DHA during parenteral and enteral nutrition in extremely premature or very low-birth-weight infants due to the need for larger quantities to compensate for intestinal malabsorption, DHA oxidation, and early deficits [34].

The early provision of breast milk, rich in DHA, promotes enhanced brain development in premature infants, supported by substantial evidence. High DHA levels in breast milk have been associated with lower incidence and severity of intraventricular hemorrhage, reduced internal capsule damage, improved white matter development, and better language and motor outcomes at 30–36 months of age. The gender-specific differentiation reveals more significant benefits for boys in terms of brain structure growth and protection against white matter damage [35,36].

## 6. Human Milk Oligosaccharides (HMOs)

Human milk contains various bioactive components with immunological functions that protect against infections, promote microbial community organization to support organ maturation, and facilitate lactocrine programming, which contributes to favorable neurodevelopmental outcomes [37]. However, a recent narrative review examining associations between exposure to Human Milk Oligosaccharides (HMO) during childhood and neurological development up to 24 months of age found limited evidence to support better neurodevelopment outcomes, particularly among premature infants [37].

Human milk oligosaccharides (HMOs) are the third most abundant solid component of human milk, following lactose and lipids. They are a group of structurally diverse complex carbohydrates, with over 150 distinct structural permutations resulting from HMO biosynthesis. The physiological functions of HMOs that influence brain maturation can vary depending on slight structural differences among them [38].

HMOs can serve as both direct and indirect sources of sialic acid, which is an essential nutrient for the organization of brain tissues. However, it is important to note that the concentration of HMOs in human milk can vary significantly based on the phenotype of the mother and the stage of lactation. Typically, HMO concentrations, relative to their abundance, are higher in colostrum compared to transitional milk and higher in transitional milk compared to mature milk [39].

Some animal studies have identified specific HMOs that may influence early brain maturation. Sialic acids (Sia), both in their free form (N-acetylneuraminic acid) and conjugated forms like 6′-sialyllactose (6′-SL), have been shown to improve cognitive abilities and memory when supplemented in rats. Recent reviews have highlighted the consistent benefits of certain HMOs, such as 2′-FL, 3-FL, 3′-SL, and 6′-SL, for improving motor skills, language development, working memory, and reference memory. However, more research through randomized and controlled clinical trials is needed to fully understand the specific mechanisms involved [40,41].

The volume of formula or human milk consumed by the preterm infant may impact the dose of HMOs, thus affecting neurological development outcomes, that is, greater the accepted volume of enteral feeding with breast milk and its HMO, and better short- and long-term outcomes.

## 7. Micronutrients

The process of myelination in the developing brain is dependent on various micronutrients, including iron, copper, iodine, vitamin B12, and choline. It is crucial to consider the timing of nutrient supplementation, especially during the critical window of oligodendroglia differentiation, which occurs between 23 and 32 weeks of gestational age when the formation of cerebral white matter is at its peak. Nutritional interventions need to coincide with the development of brain structures or circuits that rely on specific nutrients. Initiating supplementation too early or too late may not produce the desired effects [42].

Iron is essential for the function of enzymes involved in oligodendroglia differentiation and myelination. Iron deficiency is common in premature infants, and it may be associated with impaired maturation and myelination of oligodendrocytes. Studies have shown that iron-deficient neonates often exhibit motor, cognitive, and behavioral delays. Iron deficiency in neonates is often linked to nutritional deficiencies, particularly in premature infants who did not have the opportunity to accumulate sufficient iron stores during the third trimester of pregnancy. Iron’s impact on myelin formation has been confirmed through studies of auditory and visual evoked potentials. Delayed umbilical cord clamping in full-term infants has been associated with increased serum ferritin levels and myelin content at four months of age, suggesting potential benefits for myelination [43,44,45,46].

Zinc is another crucial mineral for fetal and postnatal development. It plays various roles in gene expression, cell development, replication, and the synthesis of RNA and DNA, which are essential for growth, differentiation, and cellular metabolism. Cerebellar development and NMDA receptor expression also depend on zinc. Extremely preterm infants are particularly vulnerable to zinc deficiency due to diminished stores, increased requirements, and suboptimal absorption. Zinc concentrations in human milk can vary widely. More research is needed to understand the relationship between zinc status, oligodendroglia maturation, myelination, and neurodevelopment in preterm infants [47,48,49].

## 8. Vitamins

Vitamin D is known to influence nerve growth factor, promote neurite growth, and inhibit neuronal apoptosis in the hippocampus. A deficiency during critical phases of neurodevelopment can lead to behavioral, memory, and learning disorders later in life. However, a recent systematic review with meta-analysis did not find significant evidence of improved neurodevelopment with enteral vitamin D supplementation for premature and low-birth-weight infants compared to no supplementation or a placebo [50,51].

Data are limited regarding the relationship between selected micronutrients from breast milk, such as vitamin B6, carotenoids, and selenium, and better neurodevelopmental outcomes. It is increasingly believed that a combination of nutritional factors, rather than a single nutrient, has a more significant impact on the neurodevelopment of preterm infants. A study involving British premature boys who received a high-nutrient premature formula in the first 4 weeks of life showed significantly larger volumes of the caudate nucleus at 16 years of age compared to those who received a standard full-term infant formula during the same period, highlighting the potential benefits of a comprehensive nutrient approach [52,53].

## 9. Intestinal Microbiota and the Gut-Brain Axis

The period between 24 weeks and 52 weeks of post-conceptional age is critical for neural development, characterized by significant neuronal and glial growth in the brain. During this time, the gut-brain axis plays a vital role, with the gut microbiome influencing brain functions and development. Premature infants often have immature gut microbiomes, impacting neurodevelopment. Challenges unique to premature infants include an underdeveloped intestinal barrier that fails to defend against pathogenic bacteria, leading to dysregulated responses that further compromise the immature immune system. Additionally, the underdeveloped blood–brain barrier allows for components associated with the microbiome to cross into the brain more easily, affecting brain functions [1,5,7,54].

HMOs are known to play a crucial role in establishing a healthy microbiota in early life, promoting brain and cognitive development through the gut-brain axis. Potential connections between the intestinal microbiota and the brain include modulation of the immune system, production of neurotransmitters or neuromodulators, regulation of systemic inflammation, and interaction with the Vagus nerve and blood–brain barrier [38,41,54].

Efforts to modulate the microbiome aim to prevent intestinal dysbiosis, which is characterized by an imbalance in microbial colonization. Dysbiosis can result from various exogenous factors, such as mode of delivery, formula feeding, and exposure to antibiotics. Premature infants are particularly susceptible to dysbiosis, which can lead to sepsis and necrotized enterocolitis, both of which are associated with delayed neurodevelopment. Supplementation with probiotics has shown promise in reducing the risk of these conditions and may indirectly contribute to neuroprotection through its trophic effects on the intestine [7,55].

Probiotics have the potential to be neuroprotective due to their direct effects on gene expression, neurotransmitter synthesis, expression of neurotrophic growth factors, and reduction of neuroinflammation. However, the data on the effectiveness of probiotics in premature infants are mixed, with some studies showing benefits in reducing certain risks but not consistently impacting neurocognition [54,56].

## 10. Human Milk

The advantages of breast milk for neurodevelopment are substantial and indisputable. These benefits are even influenced by the quantity of breast milk consumed. Breast milk contains docosahexaenoic acid (DHA) omega-3 fats, which, when combined with eicosapentaenoic acid (EPA) fats, can potentially reduce the risk of affective disorders, such as major depression and bipolar disorders, ultimately having a positive impact on individuals in society as they mature. While the use of human milk for feeding preterm infants has been shown to offer various neurodevelopmental advantages, other nutritional aspects that affect the growth of premature babies, including macronutrients and micronutrients, may necessitate supplementation to meet their higher nutritional requirements [1,20].

When considering consensus recommendations for feeding preterm infants, it is important to note that the first choice for feeding preterm infants is human milk expressed by their own mothers, with the second option being donated pasteurized human milk. Unpasteurized milk should not be used in the case of human donor milk [3]. The bond between mother and child has a positive association with neurodevelopmental outcomes, and this bond tends to be stronger among breastfeeding mothers, underscoring the importance of policies in neonatal intensive care units (NICUs) that promote the presence of the mother, family-centered care, and breastfeeding.

Belfort et al. observed that preterm infants born before 30 weeks’ gestation or with a birth weight less than 1250 g who predominantly received breast milk in the first 28 days of life (more than 50% of their diet) had a larger deep-nuclear-gray matter volume at term-equivalent age. Additionally, they exhibited better IQ, academic achievement, working memory, and motor function at the age of 7. The study focused on regional volumetric measurements obtained through structural magnetic resonance imaging (MRI), a non-invasive technique for studying the brain’s anatomy and pathology, distinct from functional magnetic resonance imaging (fMRI), which assesses brain activity [57].

Deoni et al. found that the composition of infant nutrition has an impact on myelin development. Their study revealed that breastfed children showed improved global cognitive ability and rates of cognitive development, including verbal and nonverbal functioning when compared to those receiving infant formula alone. Moreover, exclusive breastfeeding for at least 3 months was associated with enhanced diffuse myelination throughout the brain by the age of 2 [58].

A prior study on activation and connectivity in breastfed infants observed significantly higher gray matter volume in the left and right parietal lobes, as well as the left temporal lobe in breastfed children. Furthermore, breastfed children exhibited greater activation in the right frontal and temporal lobes during perception tasks, while for language tasks, activation was greater in the left temporal lobe [59]. It is well-established that brain activation is positively correlated with performance on cognitive tasks, further reinforcing the evidence that breastfeeding is associated with improved performance on intelligence tests. Table 2 summarizes the advantages of human milk on neurodevelopmental outcomes.

Despite numerous studies, the precise mechanisms through which the components of human milk shape the structural and functional characteristics of the infant brain remain incompletely understood. This area of research holds great promise, as children born prematurely who receive a greater supply of their own mother’s breast milk appear to exhibit higher general intelligence, improved academic performance, enhanced memory, and better motor function as they grow [16,38,41,60]. Thus, breast milk plays a pivotal role in promoting overall brain development, emerging as a key factor contributing to the positive effects of breastfeeding on intelligence.

## 11. Conclusions

Nutrition is a critical factor in achieving adequate growth and brain development in premature infants, especially those with lower gestational ages. While nutrition alone may not fully counteract the challenges of extreme prematurity, nutritional therapies hold promise for promoting brain development. These interventions, including amino acids, glucose, and LCPUFAs, are considered safe, cost-effective, and minimally invasive. They can be combined with breastfeeding, which is the optimal source of nutrition for premature infants.

In addition to the emphasis on macronutrient intake, it is crucial to consider micronutrients, neuropeptides, neurohormones, and their roles in modulating the gut-immune-brain axis. Further research is needed to explore the potential impact of donated human milk and milk pasteurization on the premature infant’s brain. Individualized nutritional approaches that address the unique trajectories of premature infants and the evolving nutritional needs are essential.

Breastfeeding may improve maternal–infant bonding, which is another possible mechanism for the positive breastfeeding association with development.

Overall, the growing recognition of human milk as the gold standard of nutrition, along with the integration of comprehensive nutrient strategies, provides hope for better outcomes in the neurodevelopment of premature infants.

## Figures and Tables

**Table 1:** Neuro-nutrition: The impact of each nutrient on the brain.

Nutrient	Impact	Brain Structure
Energy and protein	Cell multiplication and differentiation, synaptogenesis, growth factors	Cortex, hippocampus, global brain
Iron	Myelin, monoamine synthesis, glial metabolism	White matter and hippocampus
Zinc	DNA synthesis, neurotransmitters	Autonomous nervous system, hippocampus, cerebellum
Copper	Neurotransmitters, glial metabolism, antioxidation	Cerebellum
LC-PUFAS	Synaptogenesis	Retina, cortex
TAURINE/HILL	Neurotransmitters, DNA methylination, myelin	Global region, hippocampus and cerebral white matter

Adapted from [9,10].

**Table 2:** Advantages of human milk on neurodevelopment outcome.

Immediate Effects and Short Term	Long Term
Significant improvement in white matter microstructure	Better cognitive, behavioral, and academic performance
Larger deep nuclear gray matter and hippocampus volume at term-equivalent age	Improved working memory
Significantly greater brain volume and white matter volume	Significantly improved verbal IQ, especially in boys (25% increase in IQ)
Better receptive language at age 3	Better verbal and nonverbal IQ at age 7 *
Improved mental and psychomotor development scores at Bayley Scales	Significantly higher IQ in later years, even after adjustment for maternal IQ

* This result was observed in a longer duration of breast milk feeding; each month of breastfeeding can increase verbal IQ by 0.35 points and nonverbal IQ by 0.29 points. Adapted from Kumar et al. 2017 [3] and De Nardo et al. 2022 [5].
